# GM-CSF orchestrates monocyte and granulocyte responses to *Cryptococcus gattii*

**DOI:** 10.1371/journal.ppat.1013418

**Published:** 2026-04-07

**Authors:** Alison Ricafrente, Sreemoyee Acharya, Shuyi Chen, Adiza Abass, Aelita Arshakyan, Tyler J. Olson, Apurwa Trivedi, Lena J. Heung

**Affiliations:** 1 Department of Medicine, Women’s Guild Lung Institute, Cedars-Sinai Health Sciences University, Los Angeles, California, United States of America; 2 Department of Biomedical Sciences, Cedars-Sinai Health Sciences University, Los Angeles, California, United States of America; University of Notre Dame, UNITED STATES OF AMERICA

## Abstract

*Cryptococcus gattii* is an emerging fungal pathogen that is acquired through the respiratory tract and causes invasive infections in both immunocompromised and otherwise healthy people. Many of these apparently immunocompetent patients are subsequently found to have autoantibodies against the pleiotropic cytokine GM-CSF. In this study, we investigated the potential role of GM-CSF (or CSF2) in the host response to *C. gattii* using a murine model of infection. Interestingly, infected *Csf2*^*-/-*^ mice were found to have significantly improved survival and decreased lung fungal burden compared to wild type (WT) mice. We determined that during *C. gattii* infection, GM-CSF promotes the differentiation of monocytes into alveolar and interstitial macrophages. When these macrophages are ablated in CCR2-DTR^+^ mice, there is a corresponding improvement in survival with decreased lung fungal burden, thus phenocopying *Csf2*^*-/-*^ mice. WT bone-marrow derived macrophages challenged with *C. gattii* and interstitial and alveolar macrophages from infected WT mice are unable to undergo M1 polarization, suggesting that monocyte-derived macrophages (moMacs) are rendered permissive for fungal proliferation. Therefore, GM-CSF and moMacs mediate immune responses that are harmful to the host. We further found that GM-CSF and moMacs preferentially promote the influx of eosinophils over neutrophils into the infected lung which is associated with substantial inflammatory lung pathology. Ablation of neutrophils using Mrp8cre^tg^ iDTR^+^ mice significantly increased *C. gattii* burden in the lungs, indicating that GM-CSF and moMacs block the entry of these beneficial, fungal-clearing granulocytes during infection. Altogether, our results show that GM-CSF plays a key role in impeding host anti-fungal responses to *C. gattii* by coordinating monocyte-derived macrophages and granulocyte activity and crosstalk.

## Author summary

*Cryptococcus gattii* is an environmental fungus that can cause severe lung and brain infections after inhalation through the respiratory tract. *C. gattii* causes disease in patients with known immune deficits but also in individuals that are apparently healthy. Studies on otherwise healthy people who become infected with *C. gattii* suggest that they may have a previously unrecognized problem involving granulocyte macrophage-colony stimulating factor (GM-CSF), a cytokine, or messenger protein, that is an important part of the immune system. Here, we investigate the role of GM-CSF in the immune response to *C. gattii* using a mouse model of infection. We find that *C. gattii* increases GM-CSF in the lungs, leading to the influx of immune cells, including monocyte-derived macrophages and eosinophils, while inhibiting the entry of neutrophils. The macrophages and eosinophils allow the fungus to proliferate and cause inflammatory damage to the lungs, which is ultimately fatal. The absence of neutrophils also contributes to fungal growth, as these immune cells would otherwise be able to help kill the fungus. Our study provides new insight into how GM-CSF regulates immunity to *C. gattii* and has important implications as to the mechanisms that govern susceptibility to this infection.

## Introduction

*Cryptococcus gattii* is an environmental, encapsulated yeast that is an important cause of invasive lung and brain infections in humans [1]. Known to be endemic in tropical and subtropical parts of the world, *C. gattii* subsequently caused an outbreak of cryptococcosis in 1999 in the temperate regions of British Columbia and the Pacific Northwest, thus broadening its global impact [2–4]. In contrast to its opportunistic relative *Cryptococcus neoformans*, *C. gattii* can also infect apparently healthy individuals. With mortality from *C. gattii* infections estimated between 10–33% [5–7], it is critically important to understand the distinct mechanisms *C. gattii* uses to cause disease in an expanding patient population. Indeed, the World Health Organization designated *C. gattii* one of its first fungal priority pathogens in 2022 given its disease potential and the large knowledge gaps regarding its pathogenicity [8].

Studies on *C. gattii* infections in otherwise healthy people discovered a close correlation with the presence of autoantibodies (AAb) against the cytokine granulocyte macrophage-colony stimulating factor (GM-CSF or CSF2) [9–12]. Anti-GM-CSF AAb are also linked to other fungal and bacterial infections, like aspergillosis and nocardiosis, and the lung disease pulmonary alveolar proteinosis (PAP) [13–15]. GM-CSF is a cytokine that plays a critical role in the development of myeloid cells and their effector functions in a broad range of disease states, from infections to autoimmune disorders [16,17]. Although *C. gattii* infections are associated with anti-GM-CSF AAb, the role of GM-CSF in the immune response to this pathogen is not well understood.

Here, we used a fatal model of murine *C. gattii* infection to establish that GM-CSF hinders clearance of infection and promotes immunopathology in the lungs. *Csf2*^-/-^ mice that lack GM-CSF have significantly improved survival rates and decreased lung fungal burden compared to WT mice. We found that GM-CSF facilitates the differentiation of CCR2^+^Ly6C^hi^ monocytes into alveolar and interstitial macrophages and that ablating these immune cells in CCR2-DTR^+^ mice phenocopies the improved infectious outcome we observed in *Csf2*^-/-^ mice. These monocyte-derived macrophages (moMacs) have a direct role in promoting *C. gattii* proliferation because they are poorly activated when challenged with the fungus. We further determined that GM-CSF and moMacs support the pulmonary infiltration of eosinophils, that cause significant airway inflammation, while also blocking the entry of neutrophils that would otherwise be beneficial for fungal clearance. Together, these results indicate a critical role for GM-CSF in regulating a monocyte-granulocyte axis that determines host outcomes during *C. gattii* infection.

## Results

### GM-CSF mediates poor host outcomes after *C. gattii* infection

**Fig 1:**
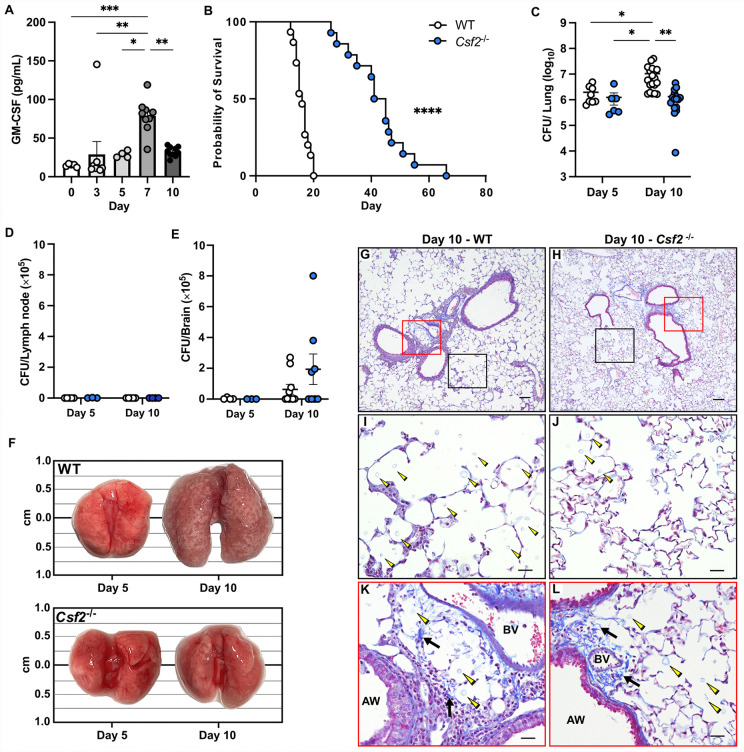
GM-CSF mediates detrimental host responses to *Cryptococcus gattii.* **(A)** Pulmonary GM-CSF cytokine levels in WT mice infected with *C. gattii*. Days 3, 7, and 10 data include *n* = 8-10 total mice per timepoint from *N* = 2 independent experiments; Days 0 (naive) and 5 data are from *n* = 5 total mice per timepoint from *N* = 1 experiment. **(B)** Kaplan-Meier survival curve of WT (white circles) and *Csf2*^*-/-*^ (blue circles) mice, *n* = 14-15 total mice per group from *N* = 3 independent experiments. **(C-E)** Fungal burden was measured in lung **(C)**, mediastinal lymph node **(D)**, and brain **(E)**. For the lung data, Day 5 includes *n* = 6-8 total mice per group from *N* = 2 independent experiments, and Day 10 includes *n* = 12-14 total mice per group from *N* = 3 independent experiments. For lymph node and brain data, Day 5 consists of *n* = 3-4 total mice per group from *N* = 1 experiment, and Day 10 consists of *n* = 8-10 total mice per group from *N* = 2 independent experiments. **(F)** Representative images of unaltered whole lungs surgically collected from WT and *Csf2*^*-/-*^ mice at Days 5 and 10 p.i. Photo credit: Alison Ricafrente, First Author. **(G-L)** Representative lung sections from WT **(G, I, K)** and *Csf2*^*-/-*^ **(H, J, L)** mice stained with Masson’s trichrome. For Panels G and H, scale bar = 100 μM at 10X magnification. The black boxes are magnified in Panels I and J to show alveolar spaces. The red boxes are magnified in Panels K and L to show the adventitial sheath. For Panels I-L, scale bar = 12 μM at 40X magnification. Fungal cells (yellow arrowheads); Airway (AW); Blood vessel (BV); Collagen fibers (black arrows). Histology images are representative of *n =* 4-5 total mice per group from *N* = 1 experiment; all fields of one lung slice from each mouse were evaluated. Data are presented as mean ± SEM and analyzed using one-way ANOVA **(A)**, Mantel-Cox test **(B)**, or two-way ANOVA **(C-E)**. *, *P* < 0.05. **, *P* < 0.01. ***, *P* < 0.001. ****, *P* < 0.0001.

To evaluate the role of GM-CSF during *C. gattii* infection, we first established a fatal, respiratory infection model by administering 10^5^ *C. gattii* strain R265 intratracheally (i.t.) to wild type (WT) C57BL/6J mice (S1 Fig). In these WT mice, total lung GM-CSF levels increased after infection, peaking at Day 7 post-infection (p.i.) (Fig 1A). When *Csf2*^-/-^ mice that lack GM-CSF were infected, they had prolonged survival with a median of 43 days as compared to 16 days for WT mice (Fig 1B). *Csf2*^-/-^ mice were able to control fungal proliferation in the lungs, while WT mice had an approximately 1 log increase in lung fungal burden between Days 5 and 10 p.i. (Fig 1C). There were no significant differences in mediastinal lymph node (MLN) or brain fungal burden (Fig 1D and 1E). Grossly, the lungs of WT mice appeared significantly abnormal compared to that of *Csf2*^-/-^ mice, with enlarged, nodular lobes evident by Day 10 p.i. (Fig 1F). On histology, WT mice had noticeably enlarged alveolar spaces at Day 10 p.i. compared to *Csf2*^-/-^ mice (Fig 1G and 1H), and these WT alveoli were more replete with fungal cells (Fig 1I and 1J). We also observed that fungal cells intercalated the collagen fibers within the adventitial sheath between bronchi and arterioles in WT mice (Fig 1K), which was not seen in *Csf2*^-/-^ mice (Fig 1L). Collectively, these data demonstrate that GM-CSF promotes fungal proliferation and invasion and distortion of lung architecture, leading to accelerated mortality rates during *C. gattii* infection.

### GM-CSF facilitates monocyte differentiation into effector cells during *C. gattii* infection

**Fig 2:**
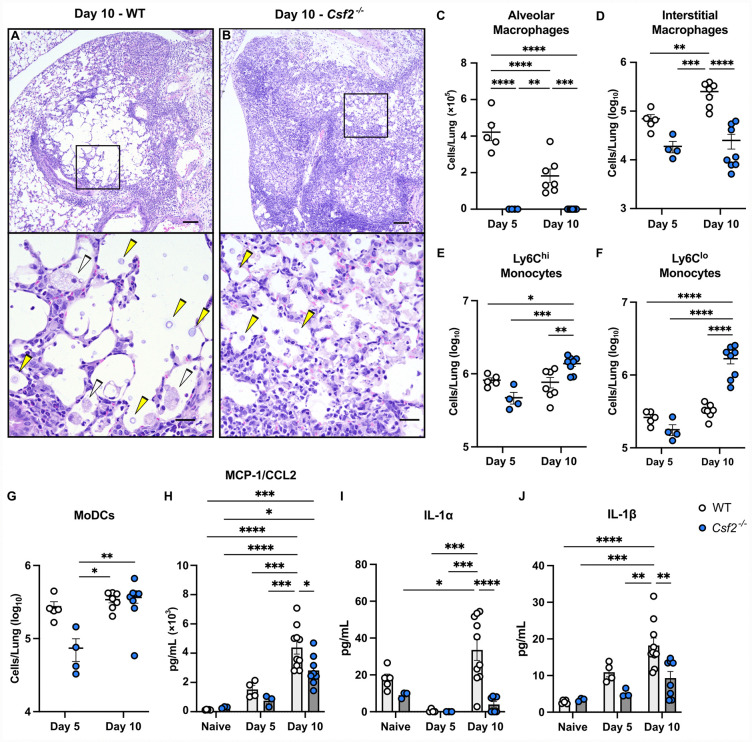
GM-CSF regulates the generation of monocyte-derived macrophages during *C. gattii* infection. **(A-B)** Representative H&E-stained lung sections of infected WT (A) and *Csf2*^*-/-*^ (B) mice at Day 10 p.i. showing pulmonary infiltrates, scale bar = 200 μM at 4X magnification. Black boxes are magnified in bottom panels, scale bar = 12 μM at 60X magnification. Fungal cells (yellow arrowhead); Foamy macrophages (white arrowhead). **(C-G)** Enumeration of lung cells from WT (white circles) and *Csf2*^*-/-*^ (blue circles) mice at Days 5 and 10 p.i., including alveolar macrophages **(C)**, interstitial macrophages **(D)**, Ly6C^hi^ monocytes **(E)**, Ly6C^lo^ monocytes **(F)**, and monocyte-derived dendritic cells (moDCs) **(G)**. **(H-J)** Pulmonary levels of cytokines MCP-1/CCL2 **(H)**, IL-1⍺ **(I)**, and IL-1β **(J)**. Histology images are representative of *n =* 4-5 total mice per group from *N* = 1 experiment; all fields of one lung slice from each mouse were evaluated. For lung cell and cytokine data, Day 5 includes *n* = 4-5 total mice per group from *N* = 1 experiment; Day 10 includes *n* = 7-10 total mice per group from *N* = 2 independent experiments. Data are presented as mean ± SEM and analyzed using two-way ANOVA **(C-J)**. *, *P* < 0.05. **, *P* < 0.01. *** *P* < 0.001. ****, *P* < 0.0001.

Since GM-CSF is known to regulate myeloid cells, we investigated what specific immune cells may be mediating downstream effects. On histology, we observed that cryptococcal cells in WT lungs were surrounded by large foamy macrophages (Fig 2A), while in *Csf2*^-/-^ lungs there was a notable lack of macrophages in similar sites (Fig 2B). *Csf2*^-/-^ mice are known to have a congenital defect in alveolar macrophage development [18,19], which we confirmed in infected *Csf2*^-/-^ versus WT mice by flow cytometry (Fig 2C). Additionally, interstitial macrophages are significantly reduced in *Csf2*^-/-^ lungs by Day 10 p.i. compared to WT (Fig 2D). During inflammation or infection, tissue resident macrophages can be supplemented by CCR2^+^Ly6C^hi^ monocytes that are recruited to the lungs and differentiate into both alveolar and interstitial macrophages [20–23]. Despite an increase in CCR2^+^Ly6C^hi^ monocytes in *Csf2*^-/-^ lungs at Day 10 p.i. (Fig 2E), there was no corresponding increase in alveolar or interstitial macrophages (Fig 2C and 2D). CCR2^+^Ly6C^lo^ monocytes, another derivative of CCR2^+^Ly6C^hi^ monocytes [24,25], are elevated in *Csf2*^-/-^ mice at Day 10 p.i. (Fig 2F), and monocyte-derived dendritic cells (moDCs) are present in comparable numbers in both mouse strains (Fig 2G). These results suggest that the lack of GM-CSF limits the differentiation of recruited monocytes into macrophages, as studies have demonstrated in other infection models [26–28]. Monocyte chemoattractant protein-1 (MCP-1) or CCL2, a chemokine that mobilizes monocytes from the bone marrow, decreases in *Csf2*^-/-^ lungs at Day 10 p.i., suggesting a backlog of monocytes in the lungs (Fig 2H) [29], and decreases in IL-1α and IL-1β in *Csf2*^-/-^ lungs (Fig 2I and 2J) may be attributable to the absence of alveolar macrophages [30]. Taken together, these studies suggest that GM-CSF regulation of monocyte differentiation into macrophages may play a key role in the immune response during *C. gattii* infection.

### Ablation of monocytes and their derivatives improves host outcomes after *C. gattii* infection

**Fig 3:**
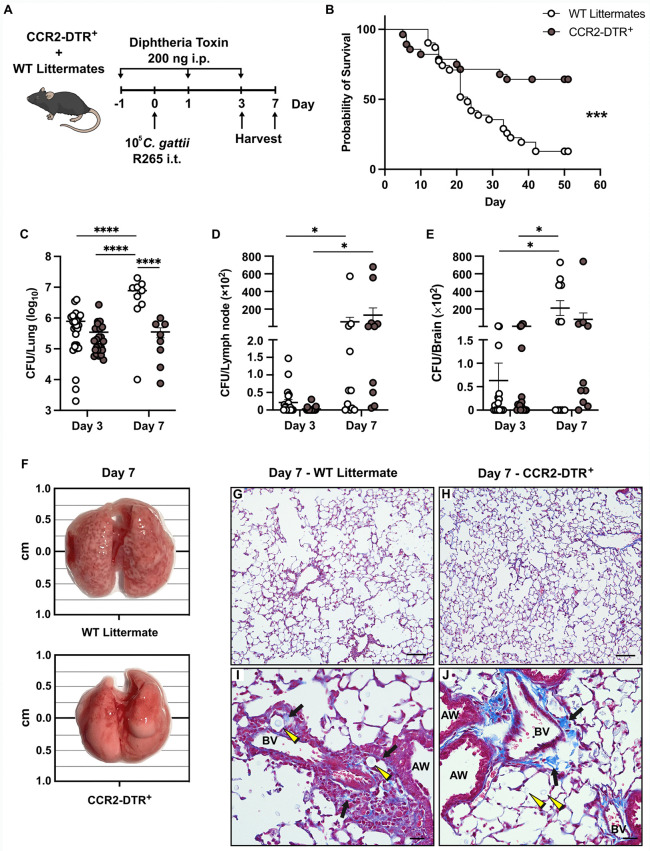
Monocytes are detrimental to the host during *C. gattii* infection. **(A)** Monocyte ablation strategy using CCR2-DTR^+^ and WT littermate mice, treated with 200 ng of diphtheria toxin by i.p. injection a day prior to infection with *C. gattii* and at Days 1 and 3 p.i. Organs were harvested at Day 3 or 7 p.i. for fungal burden measurements. **(B)** Kaplan-Meier survival curve of WT littermate (white circles) and CCR2-DTR^+^ (gray circles) mice, *n* = 28-31 total mice per group from *N* = 6 independent experiments. **(C-E)** Fungal burden was measured in lung **(C)**, MLN **(D)**, and brain **(E)**. For lungs, Day 3 includes *n* = 26-28 total mice per group from *N* = 6 independent experiments; Day 7 includes *n* = 8-9 total mice per group from *N* = 2 independent experiments. For MLN and brains, Day 3 consists of *n =* 16-17 total mice per group from *N* = 3 independent experiments; Day 7 consists of *n =* 8-9 total mice per group from *N* = 2 independent experiments. **(F)** Representative images of unaltered whole lungs surgically collected from WT littermate and CCR2-DTR^+^ mice at Day 7 p.i. Photo credit: Alison Ricafrente, First Author. **(G-J)** Representative Masson’s trichrome stain of lung sections from WT littermate and CCR2-DTR^+^ mice comparing alveoli **(G-H)**, scale bar = 100 μM at 10X magnification, and bronchovascular bundles **(I-J)**, scale bar = 25 μM at 40X magnification. Airway (AW); Blood vessel (BV); Collagen fibers (black arrows); Fungal cells (yellow arrowheads). Histology images are representative of *n =* 4 total mice per group from *N* = 1 experiment; all fields of one lung slice from each mouse were evaluated. Data are presented as mean ± SEM and analyzed using Mantel-Cox test (B) and two-way ANOVA **(C-E)**. *, *P* < 0.05. ***, *P* < 0.001. ****, *P* < 0.0001. Mouse illustration in (A) is from NIAID NIH BioArt Source (bioart.niaid.nih.gov/bioart/279).

To determine the role of monocytes and their derivative cells during *C. gattii* infection, CCR2-DTR^+^ mice [31] treated with diphtheria toxin (DT) (Fig 3A) were used to ablate CCR2^+^Ly6C^hi^ monocytes, which also resulted in the expected loss or downward trend of CCR2^+^Ly6C^lo^ monocytes, macrophages, and moDCs compared to WT littermate controls (S2A–S2E Fig). This ablation of monocytes and their derivatives improved host outcomes, as CCR2-DTR^+^ mice had a significantly improved survival rate of 64% as compared to 13% in WT mice (Fig 3B). CCR2-DTR^+^ mice also had a significant reduction in fungi in the lungs by Day 7 p.i. versus WT (Fig 3C) with no significant difference in MLN and brain fungal burden (Fig 3D and 3E). Increases in GM-CSF and MCP-1/CCL2 in the lungs of CCR2-DTR^+^ mice at Days 3 and 7 p.i. compared to WT controls suggest a feedback loop in response to the loss of CCR2^+^ monocytes and macrophages (S2F and S2G Fig); a potential source of the GM-CSF is lung epithelium [30,32,33], which should be intact in monocyte-depleted mice. Unlike *Csf2*^-/-^ mice, the reduction in pulmonary macrophages in CCR2-DTR^+^ mice did not affect the presence of IL-1 cytokines (S2H and S2I Fig), suggesting an alternative source in this model. Similar to *Csf2*^-/-^ mice, the lungs of CCR2-DTR^+^ mice appeared grossly normal at Day 7 p.i. compared to WT lungs (Fig 3F) and microscopically had less alveolar enlargement (Fig 3G and 3H) and less fungal invasion of the adventitial sheath (Fig 3I and 3J). Thus, the absence of monocytes and their derivatives improves host outcomes after *C. gattii* infection in a pattern similar to when GM-CSF is absent.

### Monocyte-derived dendritic cells do not mediate immune responses to *C. gattii*

To evaluate if moDCs play any role in determining outcomes after *C. gattii* infection, we generated CCR2-Cre^+^ MHCII^fl/fl^ mice. In this mouse model, both moDCs and conventional DCs (cDCs) lack MHCII for antigen presentation compared to MHCII^fl/fl^ littermate controls (S3A Fig and S4 Fig), as cDCs are reported to express CCR2 [34,35]. Despite the loss of MHCII expression by these DC subsets, along with a reduction in total numbers of moDCs (S3B Fig), we found no differences in survival or organ fungal burden in CCR2-Cre^+^ MHCII^fl/fl^ mice versus controls after *C. gattii* infection (S3C–S3F Fig). These data indicate that moDCs and cDCs do not play a critical role in the immune response to *C. gattii*. Thus, observed reductions in cDCs in *Csf2*^-/-^ mice [36] (S3G and S3H Fig) and in moDCs and cDCs in CCR2-DTR^+^ mice (S2E, S3I, and S3J Figs) are unlikely to have contributed significantly to the phenotypes observed. Rather, monocyte-derived alveolar and interstitial macrophages appear to facilitate the progression of disease in our model of *C. gattii* infection.

### Monocyte-derived macrophages are permissive for *C. gattii* proliferation

**Fig 4:**
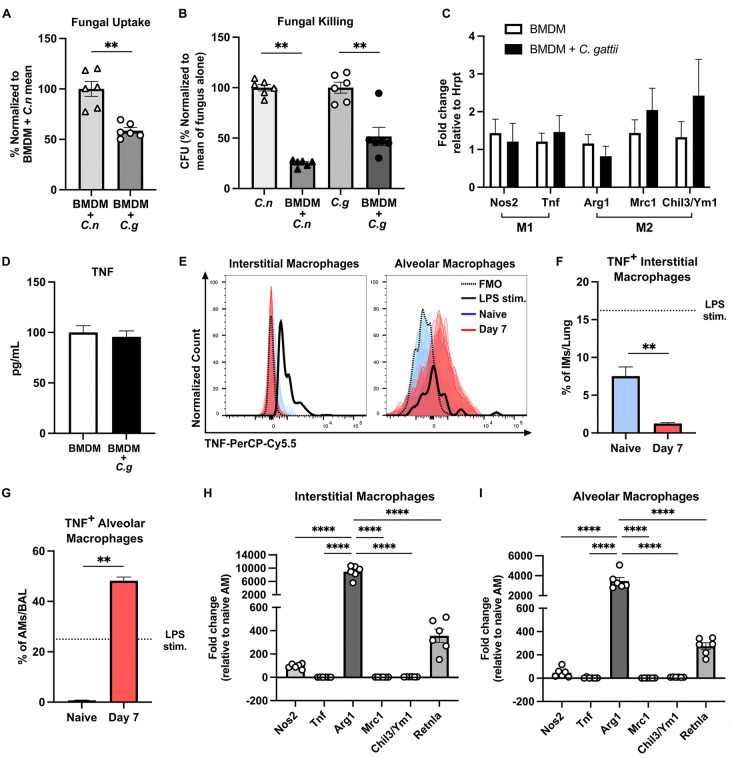
Monocyte-derived macrophages are permissive for *C. gattii* infection. **(A-D)** BMDM were challenged with 2 × 10^4^ *C. neoformans* H99-GFP or *C. gattii* R265-GFP at an MOI of 1:40 and analyzed for fungal uptake **(A)**, fungal killing **(B)**, expression of M1 and M2 polarization markers **(C)**, and TNF cytokine secretion **(D)***.* Results are from *n* = 6-9 total replicates per group from *N =* 2-3 independent experiments*.* **(E-I)** WT mice were infected with 10^5^ *C. gattii,* and on Day 7 p.i. samples from infected and naive lungs were collected for macrophage isolation. Alveolar macrophages were isolated from bronchoalveolar lavage (BAL) and interstitial macrophages were isolated from whole lung single cell suspensions. TNF expression by interstitial and alveolar macrophages from the lungs of naive (blue) or Day 7 p.i. (red) mice was measured by intracellular cytokine staining. Data are shown on a histogram **(E)**, as compared to a fluorescence minus one (FMO) control (black dotted line) and macrophages from naive mice stimulated with lipopolysaccharide (LPS) ex vivo (black line), alongside quantitation of TNF^+^ macrophages per sample **(F, G)**. The dotted line shows the percentage of macrophages from naive mice stimulated with LPS that expressed TNF **(F, G)**. RNA from macrophages was also analyzed by qRT-PCR for expression of M1 and M2 polarization markers **(H-I)**. Results are from *n* = 5 WT mice from *N* = 1 experiment. Data are presented as mean ± SEM and analyzed using Mann-Whitney test **(A-D, F, G)** and one-way ANOVA **(H,** **I)**. **, *P* < 0.01. ****, *P* < 0.0001.

To study the direct role of monocyte-derived macrophages (moMacs) in the immune response to *C. gattii*, we first challenged bone marrow-derived macrophages (BMDM) from WT mice with *C. gattii* R265-GFP or *C. neoformans* H99-GFP [37] as a comparator. We previously established that moMacs are subverted by *C. neoformans* to enable fungal proliferation in the lungs [38]. When BMDM were challenged with *C. gattii*, fungal uptake of *C. gattii* by BMDM was significantly reduced by an average of 41% relative to the uptake seen with *C. neoformans* (Fig 4A). Although there was some killing of both *C. gattii* and *C. neoformans* in the presence of BMDM, the killing of *C. gattii* by BMDM was significantly impaired*,* with only a 48% reduction in *C. gattii* colony forming units (CFUs) relative to fungus alone as compared to a 75% reduction in *C. neoformans* CFUs relative to fungus alone (Fig 4B). Interestingly, *C. gattii* does not induce significant polarization of BMDM, since there were no changes in the expression of typical markers for M1 (*Nos2* and *Tnf*) or M2 (*Arg1*, *Mrc1* and *Ym1*/*Chil3*) macrophage polarization (Fig 4C), and there was no change in TNF secretion by *C. gattii*-infected versus non-infected BMDM (Fig 4D). We also analyzed TNF production by interstitial macrophages and alveolar macrophages using intracellular cytokine staining and found that interstitial macrophages from Day 7 infected WT mice have a reduction in TNF compared to cells from naive WT mice while alveolar macrophages from infected WT mice show an increase in TNF versus naive WT cells (Fig 4E-G). However, evaluation of M1 and M2 polarization transcripts shows that both interstitial and alveolar macrophages from infected mice most highly express the M2 polarization markers *Arg1* and *Retnla* relative to naive cells (Fig 4H and 4I), indicating that *C. gattii* can impede the M1 polarization of lung macrophages. Overall, these results demonstrate that moMacs are unable to effectively activate against *C. gattii* infection, thus providing an environment that allows for fungal proliferation.

### GM-CSF and monocyte-derived macrophages induce pulmonary eosinophilia

**Fig 5:**
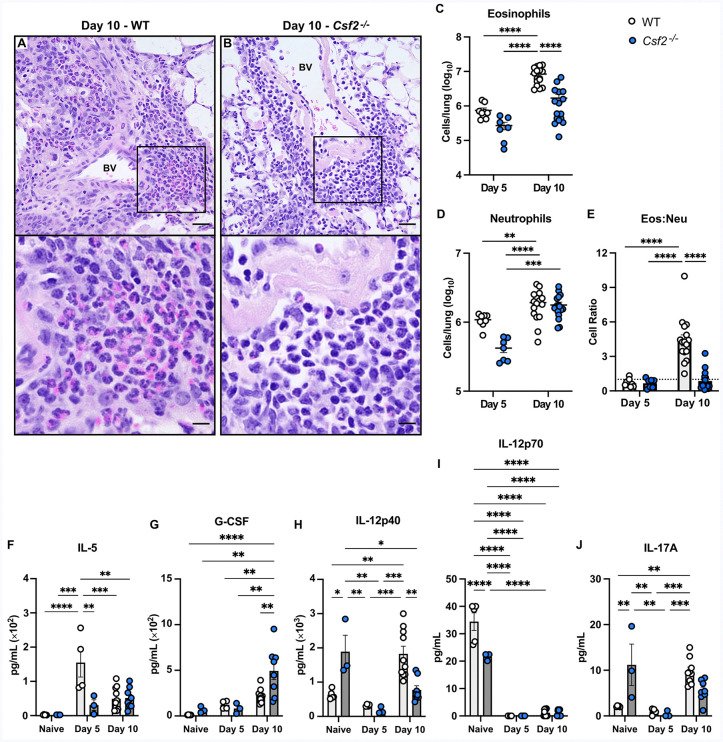
GM-CSF induces the pulmonary influx of eosinophils over neutrophils during infection. **(A-B)** Representative H&E-stained lung sections from WT **(A)** and *Csf2*^*-/-*^ **(B)** mice at Day 10 p.i. showing perivascular cell infiltrates, scale bar = 25 μM at 20X magnification. Black boxes are magnified in the bottom panels, scale bar = 12 μM at 60X magnification. BV (Blood vessel). Histology images are representative of *n =* 4-5 total mice per group from *N* = 1 experiment; all fields of one lung slice from each mouse were evaluated. **(C-E)** Flow cytometry of lung cells from WT (white circles) and *Csf2*^*-/-*^ (blue circles) mice, including eosinophils **(C)** neutrophils **(D)** and the calculated eosinophil-to-neutrophil (Eos:Neu) cellular ratio **(E)**; a ratio of 1:1 is indicated by the dotted line. Results are from *n* = 7-9 total mice per group at Day 5 p.i. from *N* = 2 independent experiments; and *n* = 16 total mice per group at Day 10 p.i. from *N* = 4 independent experiments. **(F-J)** Pulmonary levels of cytokines IL-5 **(F)**, G-CSF **(G)**, IL-12p40 **(H)**, IL-12p70 **(I)**, and IL-17 **(J)**. Naive results are from *n* = 5 total mice per group from *N* = 1 experiment; Day 5 results are from *n =* 3-4 total mice per group from *N* = 1 experiment; and Day 10 results are from *n* = 8-10 total mice per group from *N* = 2 independent experiments. Data are presented as mean ± SEM and analyzed using two-way ANOVA **(C-J)**. *, *P* < 0.05. **, *P* < 0.01. ***, *P <* 0.001. ****, *P* < 0.0001.

**Fig 6:**
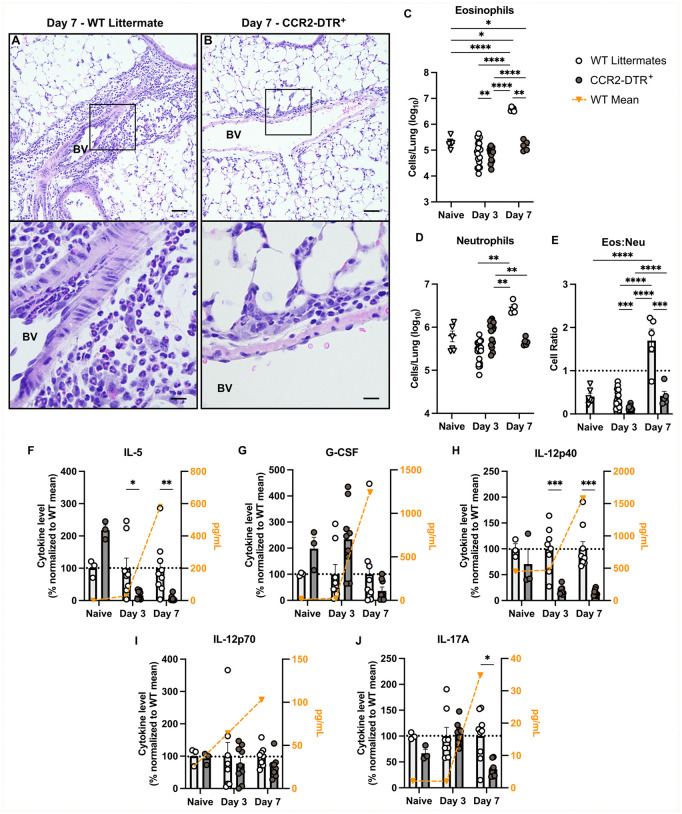
Monocytes and monocyte-derived macrophages reinforce a high eosinophil-to-neutrophil ratio in the infected lung. **(A-B)** Representative H&E-stained lung sections from WT littermates **(A)** and CCR2-DTR^+^ **(B)** mice at Day 7 p.i. showing perivascular cell infiltrates, scale bar = 25 μM at 20X magnification. Black boxes are magnified in bottom panels, scale bar = 12 μM at 60X magnification. BV (Blood vessel). Histology images are representative of *n =* 4 total mice per group from *N* = 1 independent experiment; all fields of one lung slice from each mouse were evaluated. **(C-E)** Flow cytometry of lung cells from naive WT (white triangles) and infected WT littermates (white circles) and infected *Csf2*^*-/-*^ mice (gray circles), including eosinophils **(C)**, neutrophils **(D)**, and the calculated Eos:Neu cellular ratio **(E)**; a ratio of 1:1 is indicated by the dotted line. Naive results are from *n* = 6 WT littermates from *N =* 2 independent experiments; Day 3 results are from and *n* = 15-19 total mice per group from *N* = 3 independent experiments; and Day 7 results are from *n* = 5 total mice per group from *N* = 1 experiment. **(F-J)** Pulmonary levels of cytokines IL-5 **(F)**, G-CSF **(G)**, IL-12p40 **(H)**, IL-12p70 **(I)**, and IL-17 **(J)** normalized to WT mean (black dotted line on left y-axis). Absolute WT mean cytokine levels shown by orange triangles and right y-axis. Naive results are from *n* = 3 total mice per group from *N* = 1 experiment; Day 3 results are from *n* = 8-9 total mice per group from *N* = 2 independent experiments; and Day 7 results are from *n* = 9 total mice per group from *N* = 2 independent experiments*.* Data are presented as mean ± SEM and analyzed using two-way ANOVA **(C-E)** or Mann-Whitney test **(F-J)**. Absolute WT Mean cytokine values are not included in statistical analysis. *, *P* < 0.05. **, *P* < 0.01. ***, *P <* 0.001. ****, *P <* 0.0001.

When comparing our findings in *Csf2*^-/-^ and CCR2-DTR^+^ mice, we observed similar patterns of granulocyte infiltration into the lungs, suggesting GM-CSF and moMacs may further regulate host outcomes through crosstalk with these immune cells. On lung histology there was a predominance of eosinophils in perivascular infiltrates in WT controls (Figs 5A and 6A), while in *Csf2*^-/-^ and CCR2-DTR^+^ mice there was a clear shift towards neutrophil infiltration (Figs 5B and 6B). Flow cytometry validated that total lung eosinophils were significantly higher in WT controls than in *Csf2*^-/-^ and CCR2-DTR^+^ mice (Figs 5C and 6C). The relative numbers of total lung neutrophils were more variable when comparing WT controls and *Csf2*^-/-^ and CCR2-DTR^+^ mice over time (Figs 5D and 6D). However, as infection progressed, WT controls consistently demonstrated a higher eosinophil-to-neutrophil ratio in the lungs compared to either *Csf2*^-/-^ or CCR2-DTR^+^ mice (Figs 5E and 6E). We evaluated whole lung cytokines and chemokines that might facilitate these differences in granulocyte recruitment. A key finding was that the eosinophil-associated cytokine IL-5 is significantly increased in the lungs of WT controls compared to *Csf2*^-/-^ and CCR2-DTR^+^ mice (Figs 5F and 6F). Conversely, the neutrophil-associated cytokine granulocyte-colony stimulating factor (G-CSF) is suppressed in WT lungs versus *Csf2*^*-/-*^ mice, with a similar trend comparing WT littermates to CCR2-DTR^+^ mice on Day 3 p.i. (Figs 5G and 6G). There is an increase in the cytokine subunit IL-12p40 in WT controls (Figs 5H and 6H), without any change in its Th1-associated heterodimer IL-12p70 (Figs 5I and 6I) or other Th1 and inflammatory cytokines (S2H-S2I, S5A and S6A Figs) as compared to *Csf2*^*-/-*^ and CCR2-DTR^+^ mice. WT controls demonstrated a variable amount of other Th2-associated cytokines and chemokines (S5B-S5C and S6B-S6C Figs). The Th17 cytokine IL-17 also trends higher in WT controls compared to *Csf2*^-/-^ and CCR2-DTR^+^ mice (Figs 5J and 6J). These pro-eosinophilic conditions support clear evidence of airway inflammation in WT control lungs, including goblet cell hyperplasia and metaplasia in the small and terminal airways and thickening of the subepithelial matrix with increased collagen deposition that is not present in *Csf2*^*-/-*^ and CCR2-DTR^+^ mice (S7 Fig). These findings suggest that GM-CSF and moMacs modulate cytokines and chemokines to preferentially induce pulmonary eosinophilia and inflammatory lung damage after *C. gattii* infection.

### GM-CSF and monocyte-derived macrophages suppress neutrophil influx to promote *C. gattii* proliferation

**Fig 7:**
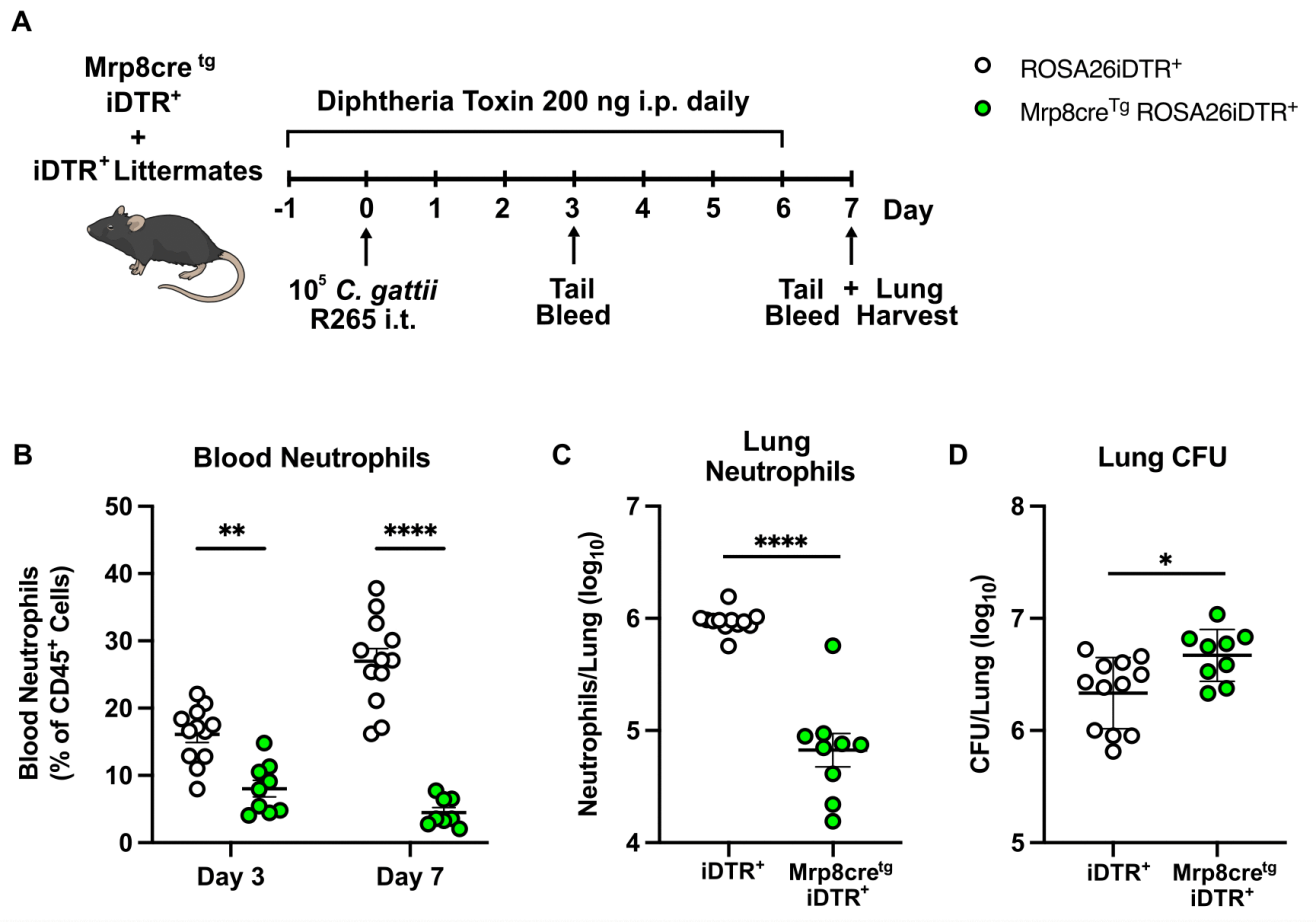
Neutrophils are beneficial to the host after *C. gattii* infection. **(A)** Neutrophil ablation strategy using Mrp8cre^tg^ iDTR^+^ mice and iDTR^+^ littermate control mice. All mice were treated with 200 ng of diphtheria toxin by i.p. injection daily, starting one day prior to i.t. infection with *C. gattii*. **(B)** At Day 3 and 7 p.i., tail vein blood was collected to measure blood neutrophils, and at Day 7 p.i., whole lungs were collected to measure total neutrophils **(C)** and fungal burden by CFU **(D)**. Results are from *n* = 9-12 total mice per group from *N* = 3 independent experiments. Data are presented as mean ± SEM and analyzed using Mann-Whitney test. *, *P* < 0.05. **, *P* < 0.01. ****, *P* < 0.0001. Mouse illustration in (A) is from NIAID NIH BioArt Source (bioart.niaid.nih.gov/bioart/279).

To determine if the reduction in pulmonary neutrophils observed in WT control mice affects progression of *C. gattii* infection, we evaluated the impact of targeted ablation of neutrophils. We initially tried antibody depletion of neutrophils with anti-Ly6G (1A8) (S7A Fig) as well as with a “Combo” protocol by Boivin et al (S7B Fig) that harnesses isotype switching to optimize neutrophil depletion [39]. These strategies achieved depletion of circulating neutrophils (S8C and S8E Fig), but there was no significant depletion of neutrophils in the lungs during *C. gattii* infection (S8D and S8F Fig). Therefore, we instead utilized Mrp8cre^tg^ iDTR^+^ mice to deplete these cells by i.p. injection of diphtheria toxin (Fig 7A) [40]. The Mrp8cre^tg^ iDTR^+^ mice were infected alongside iDTR^+^ littermate controls. We observed a sustained decrease in the percentage of total blood neutrophils in Mrp8cre^tg^ iDTR^+^ mice at Day 3 and 7 p.i. and an approximately 90% (1 log) decrease in lung neutrophils in Mrp8cre^tg^ iDTR^+^ mice at Day 7 p.i. when compared to controls (Fig 7B and 7C). This loss of neutrophils in Mrp8cre^tg^ iDTR^+^ mice resulted in a significant increase in lung fungal burden versus control mice (Fig 7D), suggesting neutrophils are beneficial for limiting *C. gattii* growth in the lungs. Thus, the absence of antifungal neutrophils in combination with the presence of permissive moMacs and pulmonary eosinophilia, all driven by GM-CSF, provides an optimal environment for fungal proliferation and lung damage, resulting in host mortality.

## Discussion

Although *C. gattii* was recognized as an emerging fungal pathogen over 25 years ago [41–43], our understanding of its interactions with the host immune system that lead to infection remains disproportionally limited. In this study, we establish that *C. gattii* induces GM-CSF signaling in the lungs, resulting in poor infectious outcomes. Our findings contrast with a recent study in which administration of recombinant murine GM-CSF led to decreased amounts of *C. gattii* in the lungs [44]. However, that study used an infection model with BALB/c mice that are more resistant to *C. gattii* than the C57BL/6J mice used in our work [44,45]. We instead found that physiologically elevated levels of GM-CSF in the lungs of C57BL/6J mice are associated with significantly increased *C. gattii* burden, immunopathology, and accelerated mortality rates. Thus, GM-CSF may play different roles in the host response to *C. gattii* in the context of different immune states, as has also been observed with *C. neoformans* [27,46–49].

Based on our studies, GM-CSF not only aids in the embryonic generation of tissue resident alveolar macrophages [18,19], but also facilitates the differentiation of recruited CCR2^+^Ly6C^hi^ monocytes into alveolar and interstitial macrophages after *C. gattii* infection. *Cryptococcus* species are facultative intracellular pathogens [1], so these moMacs may be providing a reservoir for fungal cells. Indeed, we observed that BMDM were less able to control *C. gattii* as compared to *C. neoformans*; in previous work, we established that moMacs are already very permissive for *C. neoformans* proliferation [38]. Interestingly, *C. gattii* has reduced uptake by BMDM compared to *C. neoformans*. However, clinical isolates of *C. gattii* were shown to have increased intracellular proliferation in macrophages despite lower phagocytosis rates when measured alongside *C. neoformans* [50]. BMDM also do not seem to polarize significantly and demonstrate no change in TNF secretion in response to *C. gattii*. A previous study using J774.16 macrophages, a murine cell line, have highlighted the immune silencing capacity of *C. gattii* that includes inhibition of genes associated with macrophage autophagy and polarization [51]. In vivo, we found that interstitial and alveolar macrophages from infected WT mice exhibit a strong transcriptional profile for M2 activation compared to macrophages from naive WT mice. Together, these data indicate that *C. gattii* can prevent M1 polarization of moMacs generated under the direction of GM-CSF, thereby facilitating further proliferation of the fungus.

**Fig 8:**
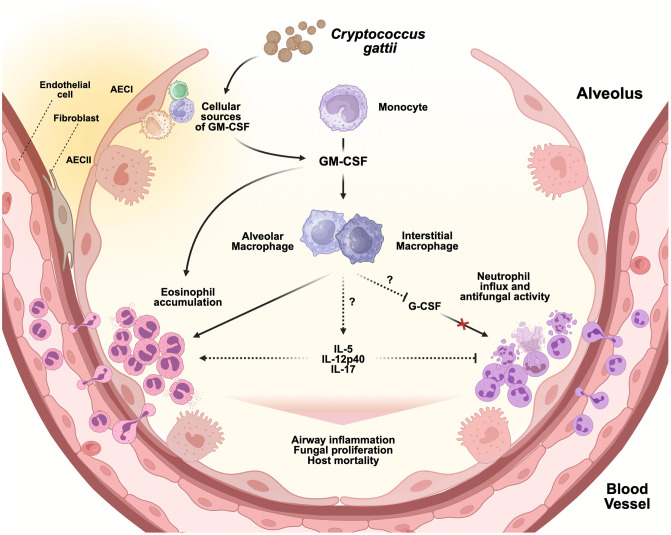
Schematic model of GM-CSF and monocyte signaling during *Cryptococcus gattii* infection. After *C. gattii* enters the lungs, the fungus may induce various cells, such as Type I (AECI), Type II alveolar epithelial cells (AECII), endothelial cells, or fibroblasts to release GM-CSF. This GM-CSF induces recruited CCR2^+^Ly6C^hi^ monocytes to differentiate into alveolar and interstitial macrophages. However, these macrophages cannot undergo M1 polarization, which then enables *C. gattii* to proliferate and invade host tissues unchecked. GM-CSF and monocyte-derived macrophages also facilitate the pulmonary influx of eosinophils while inhibiting the entry of neutrophils through the regulation of cytokines including IL-5, G-CSF, IL-12p40, and IL-17. The accumulation of eosinophils leads to harmful airway inflammation. The absence of anti-fungal neutrophils allows further fungal proliferation. Altogether, the secretion of GM-CSF and the generation of monocyte-derived macrophages fuel an imbalance of eosinophils and neutrophils that ultimately results in host mortality. Created in BioRender. Ricafrente, A. (2025) https://BioRender.com/lpowwec.

Granulocytes also appear to have a significant role downstream of GM-CSF and MoMacs during infection. The worse infectious outcomes in WT mice, as compared to both *Csf2*^-/-^ and CCR2-DTR^+^ mice, are associated with an increase in eosinophils and a relative decrease in neutrophils in the lungs. Eosinophils have well-established roles in a wide range of inflammatory pulmonary disorders like asthma and COPD [52–54]. Accordingly, the pulmonary eosinophilia we see in WT mice is associated with significant airway remodeling including goblet cell hyperplasia and metaplasia and collagen deposition in a thickened subepithelial matrix, similar to that described in asthma [55,56]. GM-CSF is known to promote the accumulation and survival of eosinophils in allergic airway inflammation [57], and macrophages can play a role in recruiting eosinophils to peripheral tissues [58]. We also see increases in other cytokines in WT mice that can promote eosinophils, including IL-5, IL-12p40, and IL-17. IL-5 has established roles in supporting the development, accumulation, and activity of eosinophils [57,59]. IL-12p40 can be secreted by macrophages and monocytes [60], and as a free monomer or homodimer, IL-12p40/IL-12p80 can act as an antagonist of IL-12 signaling [61–64]. IL-12 signaling could otherwise induce apoptosis by eosinophils and promote antimicrobial neutrophil activity [65,66]. IL-17 (or IL-17A) is generally known to drive neutrophil responses [67], but it can also induce pulmonary eosinophilia during allergic aspergillosis [68] and be further secreted by eosinophils themselves [69]. The reduction of neutrophils in the infected WT lung is likely due, in part, to the observed decreased levels of G-CSF, a cytokine that typically promotes neutrophil migration and activity in peripheral tissues, in addition to its role in granulopoiesis [70]. This lack of neutrophil influx is important, as we demonstrate that neutrophil-depleted mice are less able to control *C. gattii* proliferation in the lungs. The difference in lung fungal burden between control and neutrophil-depleted mice is significant but modest; this could be due to technical limits on how completely neutrophils can be ablated in this system or due to GM-CSF-mediated immune mechanisms, including the ongoing presence of macrophages, that may temper the overall effect. However, previous in vitro studies have established that mouse and human neutrophils have direct fungicidal activity against *C. gattii* [44,71]. In sum, GM-CSF may have direct and indirect roles, via moMacs and other immune cells, in the control of granulocyte influx and subsequent immunopathology and fungal proliferation during *C. gattii* infection (Fig 8).

We recognize that our study has only addressed some facets of how GM-CSF may regulate *C. gattii* infection, and it remains to be investigated if these mechanisms are recapitulated in human models. Although GM-CSF is linked to beneficial host responses in other fungal diseases [27,32,46–48,72–74], there is some precedent for GM-CSF increasing susceptibility to infections. In particular, pulmonary GM-CSF has been linked to worse clinical outcomes for severe COVID-19 due to its role in dysregulation of myeloid cells [75,76]. This observation has led to trials on the use of anti-GM-CSF monoclonal antibodies as potential treatments for COVID [77,78]. While our work did not directly study the role of anti-GM-CSF AAb, our findings challenge the prevailing idea that these autoantibodies may increase susceptibility to *C. gattii* [12,79]. Thus, it remains a possibility that anti-GM-CSF AAb do not play a pathogenic role in cryptococcosis, which could explain why their serum levels or degree of neutralization activity do not seem to have a direct correlation to the development of disease [12,80]. Rather, the induction of GM-CSF by *C. gattii* may set up conditions for autoimmunization, resulting in the development of anti-cytokine autoantibodies which can be a physiologic process in healthy people to help maintain homeostasis [81,82]. We also note that although the phenotypes of *Csf2*^-/-^ and CCR2-DTR^+^ mice are very similar, some features do not fully overlap and, thus, indicate the presence of mechanisms that may be regulated by GM-CSF or monocytic cells independently. For example, CCR2-DTR^+^ mice exhibit sustained survival during the observation period while *Csf2*^-/-^ mice only have delayed mortality, suggesting monocytes and their derivatives have a more encompassing impact on infectious outcomes than GM-CSF alone. Additionally, CCR2-DTR^+^ mice had more profound decreases in Th2-associated cytokine responses in the lungs as compared to *Csf2*^-/-^ mice. Future work using targeted, cell-specific approaches will help to further define the attributable downstream effects of GM-CSF versus monocytes and to identify additional cells and signaling molecules that may facilitate their roles in host immunity.

While we still have much to learn about how *C. gattii* causes disease, our study provides additional insight into the key cell types and signaling pathways that are critical to host susceptibility. We have established a new role for GM-CSF signaling in the pathogenesis of *C. gattii* through its regulation of moMacs and crosstalk with granulocytes during cryptococcosis. This work lays a foundation for further investigation of GM-CSF, monocytes, and moMacs as potential immunomodulatory targets to reduce mortality and other complications from *C. gattii* infection.

## Materials and methods

### Ethics statement

All animal procedures were performed with approval by the Institutional Animal Care and Use Committee (IACUC) at Cedars-Sinai Medical Center under protocol 8429 and were compliant with all applicable provisions established by the Animal Welfare Act and the Public Health Services Policy on the Humane Care and Use of Laboratory Animals.

### Preparation of cryptococcus

*C. gattii* strain VGIIa R265 (MYA-4093) was obtained from ATCC. *C. neoformans* strain H99 #4413 was kindly provided by Joseph Heitman (Duke University). Fluorescent strains R265-GFP and H99-GFP were a generous gift from Robin May (University of Birmingham) [37]. All *Cryptococcus* strains were grown on Sabouraud dextrose agar (SAB) plates from frozen glycerol stocks and then cultured overnight at 37°C in YPD broth (1% yeast extract, 2% peptone, 2% dextrose). Fungal cells were washed three times with sterile phosphate-buffered saline (PBS) and resuspended in PBS for further use.

### Mice

Male and female mice were used at 6–8 weeks of age, unless otherwise noted. Mice in experimental and control groups were age- and sex-matched for each experiment. C57BL/6J (strain #000664), B6.129X1-*H2-Ab1*^*b-tm1Koni*^/J (strain #013181, referred to as MHCII^fl/fl^), and *Csf2*^*-/-*^ (strain #026812) mice were purchased from Jackson Laboratory. MRP8-Cre-ires/GFP^+^ ROSA26iDTR^+^ mice (referred to as Mrp8cre^Tg^ iDTR^+^ mice) were generated using Jackson strains #021614 and #007900 and were generously provided by Keith Chan (Houston Methodist). The CCR2-DTR^+^ and CCR2-Cre^+^ mice were generated as previously described [31,38]. All mouse strains were bred and housed in the Cedars-Sinai Medical Center vivarium under specific pathogen-free conditions. Experiments using CCR2-DTR^+^, CCR2-Cre^+^ MHCII^fl/fl^, and Mrp8cre^Tg^ iDTR^+^ mice included littermate control mice that were weaned from the same litters and co-housed. The genotypes of CCR2-DTR^+^ mice were validated by the presence of CD45^+^CD11b^+^CFP^+^ cells in tail vein blood samples using flow cytometry as previously described [31]. The genotypes of Mrp8cre^Tg^ mice were validated by the presence of CD11b^+^Ly6G^+^GFP^+^ cells in tail vein blood samples using flow cytometry. The genotypes of CCR2-Cre^+^, MHCII^fl/fl^, and ROSA26iDTR^+^ (referred to as iDTR^+^) mice were validated by PCR of ear DNA as previously described [38] or as per Jackson Laboratory protocol.

### Murine infection with *Cryptococcus gattii*

Overnight YPD broth cultures of *C. gattii* R265 were washed three times with sterile PBS and resuspended at a concentration of 10^5^ cells per 50 µL volume. Mice were anesthetized with inhaled isoflurane and 50 µL of the fungal cell suspension was administered intratracheally (i.t.) using a blunt ended 20-gauge dispensing tip, as previously described [83]. For survival experiments, mice were monitored for signs of illness and euthanized as per IACUC protocol. For all other readouts, timepoints were chosen for each model based on the survival curves, in order to acquire data over equal time intervals during the course of infection.

### Immune cell ablation

To ablate monocytes and neutrophils, respectively, CCR2-DTR^+^ and Mrp8cre^Tg^ iDTR^+^ mice with their control littermates were injected intraperitoneally (i.p.) with 200 ng (10 ng/g body weight) of diphtheria toxin (List Biological Laboratories) with the frequency indicated in Figs 3A and 7A. To deplete neutrophils using antibody-based methods, mice were either given 1) anti-mouse Ly6G (clone 1A8, BioXCell) intraperitoneally or rat IgG2a isotype control (clone 2A3, BioXCell) intratracheally (S8A Fig), or 2) anti-mouse Ly6G plus mouse anti-rat kappa Ig light chain (clone MAR 18.5, BioXCell) or rat IgG2a isotype control plus mouse anti-rat kappa Ig light chain intraperitoneally (S8B Fig) [39]. Neutrophils were measured in blood and lung using flow cytometry to identify CD45^+^Ly6B.2^+^F4/80^-^Ly6C^lo^CD11b^+^ cells.

### Fungal burden in organs

After infection, whole organs including lungs, mediastinal lymph nodes (MLN), and brains were collected from euthanized mice into sterile PBS. Lungs and brain were homogenized in gentleMACS C tubes using a gentleMACS Octo Dissociator (Miltenyi Biotec). Specifically, lung samples were homogenized in 5 mL PBS + 0.5% Bovine Serum Albumin (BSA) (Fisher Scientific) + 2.31 mg/mL Collagenase Type 4 (Worthington Biochemical Corporation) + 100 mg/mL DNAse I grade II (Roche) using the “Mouse Spleen 1” program, incubated at 37°C for 45 min with gentle rotation, and further homogenized using the “Mouse Lung 2” program. Brain samples were homogenized in 1 mL PBS using the “Mouse Lung 2” program. MLN were mechanically dissociated using ground glass slides in 1 mL PBS. Tissue homogenates were serially diluted in PBS and cultured on SAB plates at 37°C. After 3 days, fungal colonies were counted and total colony forming units (CFU) per organ were calculated.

### Cytokine and chemokine measurement

To analyze cytokine and chemokine levels, whole mouse lungs were collected into 2 mL PBS containing 1X Halt Protease Inhibitor Cocktail (Thermo Fisher Scientific) followed by mechanical tissue dissociation using gentleMACS C tubes and the “RNA 1” program on a gentleMACS Octo Dissociator (Miltenyi Biotec). Tissue homogenates were centrifuged at 1500 rpm to remove cell debris, and the supernatant was collected. Supernatants were processed using the Bio-Plex Pro Mouse Cytokine 23-Plex Assay (Bio-Rad) and analyzed on a Bio-Plex 200 System (Bio-Rad). Naive samples from WT and *Csf2*^-/-^ mice and from WT littermate and CCR2-DTR^+^ mice were collected and analyzed for cytokine levels independently from infected samples. To represent Day 0 naive mice, WT littermate and CCR2-DTR^+^ mice were given one dose of 200 ng diphtheria toxin i.p., and samples were collected one day later (see Fig 3A for reference).

### Flow cytometry

To collect cells by bronchoalveolar lavage (BAL), 2 mL PBS was used to flush the airways of euthanized mice using a syringe attached to an 18G IV catheter that was inserted into a tracheal incision, as previously described [83]. For whole lungs, single cell suspensions were generated using mechanical tissue dissociation and digestion as described in the “Fungal burden in organs” section of the Materials and Methods. Isolated BAL or whole lung cells were treated with 1X RBC lysis buffer for 5 min and then washed with 0.5% BSA in PBS. Whole lung cell suspensions were passed through a 100 µM cell strainer. Total cells were counted using a hemocytometer. Cells were then stained with fluorescent antibodies against cell surface antigens (S1 Table). To isolate macrophages, an EasySep Mouse F4/80 Positive Selection Kit (STEMCELL Technologies) was used to process lung and BAL preparations; some of the isolated macrophages were challenged with 100 ng/mL lipopolysaccharide (LPS) (Sigma-Aldrich) for 2 hrs in RPMI with 10% FBS and 0.05 mM 2-mercaptoethanol at 35°C and 5% CO_2_. Macrophage samples that were used for intracellular cytokine staining (ICS) were first incubated with Cell Stimulation Cocktail (Thermo Fisher Scientific) for 4 hours at 37°C and 5% CO_2_ in complete RPMI media (10% FBS, 100 µg/mL penicillin/streptomycin, 10mM HEPES, 50 mM β-mercaptoethanol), and then processed for cell surface staining followed by fixation and permeabilization using the Cytofix/Cytoperm kit (BD Biosciences) for 30 min at 4°C. Subsequently, macrophages were washed twice with Perm/Wash buffer (BD Biosciences) and stained with anti-TNFα (PerCP Cy5.5 Cat # 506321 Biolegend or FITC Cat # 506303 Biolegend) 1/100 diluted in Perm/Wash solution for 30 min at 4°C. After washing twice, cells were kept in 0.5% BSA in PBS until acquisition. Flow cytometry data was collected on a LSRFortessa (BD Biosciences) and analyzed with FlowJo software v10.10. Gating strategies are shown in S4, S9, and S10 Figs.

### Histopathology

The lungs of euthanized mice were instilled with 4% paraformaldehyde (PFA) in PBS *in situ* via a catheter inserted through an incision in the trachea. The lungs were then harvested and fixed by immersion in 4% PFA overnight and stored in 70% ethanol until processing. Lungs were embedded in paraffin using an ASP300S tissue processor (Leica), and 5 µm sections were generated using a Microm HM 325 microtome (Thermo Fisher Scientific). Lung sections were stained with StatLab Masson’s Trichrome stain kit (Fisher Scientific) or Harris Hematoxylin Solution, Modified (Millipore Sigma) and Eosin Y solution, Alcoholic (Millipore Sigma) (H&E). Images were captured using a Nikon Ti2 microscope. Figure images shown of WT and *Csf2*^-/-^ stained lung sections at Day 10 p.i. represent *n* = 4–5 mice per group from *N* = 1 experiment. Figure images shown of WT littermate and CCR2-DTR^+^ stained lung sections at Day 7 p.i. represent *n* = 5 mice per group from *N* = 1 experiment. All fields of one lung slice from each mouse were evaluated to determine the reported findings.

### Bone-marrow derived macrophage studies

Bone-marrow derived macrophages (BMDM) were generated from C57BL/6J mouse bone marrow using L929 cell-conditioned medium, as previously described [84]. Freshly cultured R265-GFP and H99-GFP cells were opsonized for 1 hour at room temperature with murine anti-glucuronoxylomannan monoclonal antibody 18B7, kindly provided by Arturo Casadevall (Johns Hopkins), at a concentration 10 ug/mL in DMEM media. Opsonized fungal cells were used to challenge BMDM at a multiplicity of infection (MOI) of 1:40 for 24 hours at 37°C and 5% CO_2_. Killing of fungal cells by BMDM was measured by plating CFU from serial dilutions of BMDM lysed in water. Uptake of the fluorescent fungal cells by BMDM was measured by flow cytometry analysis on an LSRFortessa (BD Biosciences). Mouse TNF cytokine secretion was measured by ELISA (Invitrogen) on cell culture supernatant using a Varioskan Lux Multimode Microplate Reader (Thermo Fisher Scientific). For quantitative RT-PCR, total RNA was extracted from BMDM using TRIzol LS (Thermo Fisher Scientific), and cDNA was generated using a High Capacity RNA to cDNA Kit (Applied Biosystems). The cDNA was inspected and normalized using a Nanodrop Spectrophotometer (Thermo Fisher Scientific), and qRT-PCR was performed on a ViiA 7 Real-Time PCR System (Applied Biosystems) using TaqMan Fast Advanced Master Mix and TaqMan Gene Expression Assays (Thermo Fisher Scientific), including Arg1 (Mm00475988_m1), Mrc1 (Mm01329362_m1), Retnla/Fizz1 (Mm00445109_m1), Hprt (Mm03024075_m1), Nos2 (Mm00440502_m1), Tnf (Mm00443258_m1), and Chil3/Ym1 (Mm00657889_mH). Relative expression of transcripts was normalized using Hprt as a housekeeping gene.

### Statistical analysis

All results were expressed as mean ± SEM. A Mann-Whitney U test was used for statistical analysis of two group comparisons. One-way ANOVA was used for comparisons of 3 or more groups with one independent variable, and two-way ANOVA was used for comparisons of 3 or more groups with more than one independent variable, unless otherwise noted. Survival data was analyzed by Mantel-Cox test. All statistical analyses were performed with GraphPad Prism software, v10.2.0. A *P* value < 0.05 was considered significant and indicated with an asterisk.

## Supporting information

S1 Fig Kaplan-Meier survival curve of wild type mice infected with different inocula of *Cryptococcus gattii* R265 intratracheally. Experiments were terminated on Day 61 post-infection. Data are from *n* = 6–7 total mice per group from *N* = 2 independent experiments. *, *P* < 0.05. ***, *P* < 0.001.
(TIF)

S2 Fig Effects of monocyte ablation on derivative cells and pulmonary cytokines. (A-E) Flow cytometry of lung cells from naive and infected WT littermates and infected CCR2-DTR^+^ mice, including Ly6C^hi^ monocytes (A), Ly6C^lo^ monocytes (B), alveolar macrophages (C), interstitial macrophages (D), and moDCs (E). Data include *n* = 6 total naive mice from *N =* 2 independent experiments; Day 3 is *n* = 15–19 total mice per infected group from *N* = 3 independent experiments; and Day 7 is *n* = 5 total mice per infected group from *N* = 1 experiments. (F-G) Pulmonary levels of cytokines GM-CSF (F), MCP-1/CCL2 (G), IL-1α (H), and IL-1β (I) normalized to WT mean (black dotted line on left y-axis). WT mean cytokine levels shown by orange triangles and right y-axis. Results are from *n* = 3 total naive mice per group from *N* = 1 experiment, *n* = 8–9 total mice per group at Day 3 p.i. from *N* = 2 independent experiments, and *n* = 9 total mice per group at Day 7 p.i. from *N* = 2 independent experiments. Data are presented as mean ± SEM and analyzed using two-way ANOVA (A-E) or Mann-Whitney test (F-I). Absolute WT Mean cytokine values are not included in statistical analysis. *, *P* < 0.05. **, *P* < 0.01. ***, *P* < 0.001. ****, *P* < 0.0001.
(TIF)

S3 Fig Dendritic cells do not have a role in the response to *C. gattii* infection. (A-B) Flow cytometry of dendritic cells from the lungs of MHCII^fl/fl^ controls (black line/white circles) and CCR2-Cre^+^ MHCII^fl/fl^ mice (orange filled line/orange circles) at Day 7 p.i., including a histogram of MHCII expression (A) and total numbers (B) of CD11b^+^ conventional DC (cDC), CD103^+^ cDC, and monocyte-derived DCs (moDCs). Data are from *n* = 8 total mice per group from *N =* 2 independent experiments. (C) Kaplan-Meier survival curve of MHCII^fl/fl^ and CCR2-Cre^+^ MHCII^fl/fl^ mice, *n* = 8–10 total mice per group from *N* = 2 independent experiments. (D-F) Fungal burden was measured from lung (D), MLN (E), and brain (F) at Day 7 p.i. Data are from *n* = 8 total mice per group from *N =* 2 independent experiments. (G-H) Flow cytometry of lung cells including CD11b^+^ cDC (G) and CD103^+^ cDC (H) from WT and *Csf2*^*-/-*^ mice at Days 5 and 10 p.i. Results are from *n* = 5 total mice per group at Day 5 p.i. from *N* = 1 experiment, and *n =* 7–8 total mice at Day 10 p.i. from *N =* 2 independent experiments. (I-J) Flow cytometry of lung cells from naive and infected WT littermates and infected CCR2-DTR^+^ mice at Days 3 and 7 p.i., including CD11b^+^ cDC (I) and CD103^+^ cDC (J). Results are from *n* = 6 naive WT littermate mice from *N =* 2 independent experiments and *n* = 5 total infected mice per group from *N* = 1 experiment. Data are presented as mean ± SEM and analyzed using Mann-Whitney test (B, D-F), Mantel-Cox test (C), or two-way ANOVA (G-J). ns, not significant. *, *P* < 0.05. **, *P* < 0.01. ***, *P* < 0.001. ****, *P* < 0.0001.
(TIF)

S4 Fig Gating strategy for DCs in CCR2-Cre^+^MHCII^fl/fl^ and MHCII^fl/fl^ mice. Lung cells from MHCII^fl/fl^ littermate controls were collected for flow cytometry analysis on Day 7 p.i. This strategy first excludes SiglecF^+^, MerTK^+^, CD3^+^, and CD19^+^ cells and then separates monocyte-derived and non-monocyte-derived cells by CD64 histogram. FSC-A^hi^ cells were selected for CD11b^+^ vs CD103^+^ gating. CD11b^+^ and CD103^+^ cDCs and moDCs were then determined by using a MHCII expression overlay to determine MHCII^+^ cell populations in the littermate control mice. These overlays were then applied to DC gates in CCR2-Cre^+^ MHCII^fl/fl^ samples.
(TIF)

S5 Fig Pulmonary Th1 and Th2 cytokines and chemokines in WT versus *Csf2*^-/-^ mice. (A-C) Levels of pulmonary cytokines in naive infected WT and *Csf2*^*-/-*^ mice including Th1-associated and inflammatory cytokines (A) and Th2-associated cytokines (B) and chemokines (C). Results are from *n* = 5 total naive mice per group from *N* = 1 experiment, *n =* 3–4 total mice per group at Day 5 p.i. from *N* = 1 experiment, and *n* = 8–10 total mice per group at Day 10 p.i. from *N* = 2 independent experiments. Data are presented as mean ± SEM and analyzed using two-way ANOVA. *, *P* < 0.05. **, *P* < 0.01. ***, *P* < 0.001. ****, *P* < 0.0001.
(TIF)

S6 Fig Pulmonary Th1 and Th2 cytokines and chemokines in WT littermate versus CCR2-DTR^+^ mice. (A-C) Levels of pulmonary cytokines in infected WT littermate and CCR2-DTR^+^ mice including Th1-associated and inflammatory cytokines (A), Th2-associated cytokines (B), and chemokines (C) normalized to WT mean (black dotted line on left y-axis). WT mean cytokine levels shown by orange triangles and right y-axis. Results are from *n* = 3 total naive mice per group from *N* = 1 experiment, *n* = 8–9 total mice per group at Day 3 p.i. from *N* = 2 independent experiments, and *n* = 9 total mice per group at Day 7 p.i. from *N* = 2 independent experiments. Data are presented as mean ± SEM and analyzed using Mann-Whitney test. WT mean cytokine values are not included in statistical analysis. *, *P* < 0.05. **, *P* < 0.01. ***, *P* < 0.001.
(TIF)

S7 Fig GM-CSF and monocyte-derived macrophages promote airway inflammation. (A-B) Representative Masson’s trichrome-stained sections showing large airways, small airways, and terminal airways in the lungs of WT and *Csf2*^*-/-*^ mice at Day 10 p.i. (A) and WT littermate and CCR2-DTR^+^ mice at Day 7 p.i. (B). Images in the left panels for each mouse strain are shown with scale bar = 100 μM at 10X magnification. Black boxes are magnified in the right panels for each mouse strain, with scale bar = 12 μM at 60X magnification. Goblet cells (green arrowheads); subepithelial layer (orange arrows). Histology images are representative of *n =* 4–5 total mice per group from *N* = 1 experiment; all fields of one lung slice from each mouse were evaluated.
(TIF)

S8 Fig Antibody-based neutrophil depletion in C57BL/6J mice during *C. gattii* infection. (A-B) C57BL/6J mice infected with 10^5^ *C. gattii* i.t. were treated with anti-mouse Ly6G or IgG2a isotype control given both i.t. and i.p. daily (A) or treated with anti-rat κ Ig light chain i.p. on the days indicated plus anti-mouse Ly6G or IgG2a isotype control by i.p. daily (B). Tail bleeds at Day 3 and Day 7 p.i. were performed to measure the percentage of blood neutrophils against CD45^+^ cells by flow cytometry (C, E). Whole lungs were harvested at Day 7 p.i. for measurement of total neutrophils by flow cytometry (D, F). Results are from *n* = 4–8 total mice per group per time point from *N* = 1 experiment. Data are presented as mean ± SEM and analyzed using Mann-Whitney test (C-F). *, *P* < 0.05. ***, *P* < 0.001. Mouse illustrations in (A and B) are from NIAID NIH BioArt Source (bioart.niaid.nih.gov/bioart/279).
(TIF)

S9 Fig Gating strategy for macrophage, monocyte, and granulocyte cell populations in murine lungs by flow cytometry. Lung cells from a naive CCR2-DTR^+^ mouse were collected for flow cytometry analysis. Gating strategy for macrophages (CD45^+^CD64^+^MerTK^+^) including alveolar macrophages (CD11b^−^CD11c^hi^SiglecF^+^) and interstitial macrophages (CD11b^+^CD11c^+^SiglecF^−^); monocytes (CD45^+^CD64^+^MerTK^−^CD11c^−^SiglecF^-^CD11b^+^) including Ly6C^hi^ monocytes and Ly6C^lo^ monocytes; and granulocytes (CD45^+^CD64^−^MHCII^-−^CD11c^−^CD11b^+^SSC^hi^) including neutrophils (Ly6G^+^SiglecF^−^) and eosinophils (Ly6G^−^SiglecF^+^).
(TIF)

S10 Fig Gating strategy for dendritic cell subsets in murine lungs by flow cytometry. Lung cells from a naive CCR2-DTR^+^ mouse were collected for flow cytometry analysis. This strategy first excludes SiglecF^+^, MerTK^+^, CD3^+^, and CD19^+^ cells and then identifies DCs (CD45^+^CD3^−^CD19^−^MerTK^−^SiglecF^−^ MHCII^hi^ CD11c^+^) including CD11b^+^ cDCs (CD64^−^CD11b^+^CD103^−^), CD103^+^ cDCs (CD64^−^ CD11b^−^CD103^+^), and moDCs (CD64^+^CD11b^+^ CD103^−^).
(TIF)

S1 Table Conjugated antibodies used for flow cytometry experiments. (TIF)
