# Supplementary figures and images for: GM-CSF orchestrates monocyte and granulocyte responses to Cryptococcus gattii
Source: PLoS Pathog. 2026 Apr 7;22(4):e1013418. doi: 10.1371/journal.ppat.1013418 (PMC13068330; doi:10.1371/journal.ppat.1013418)

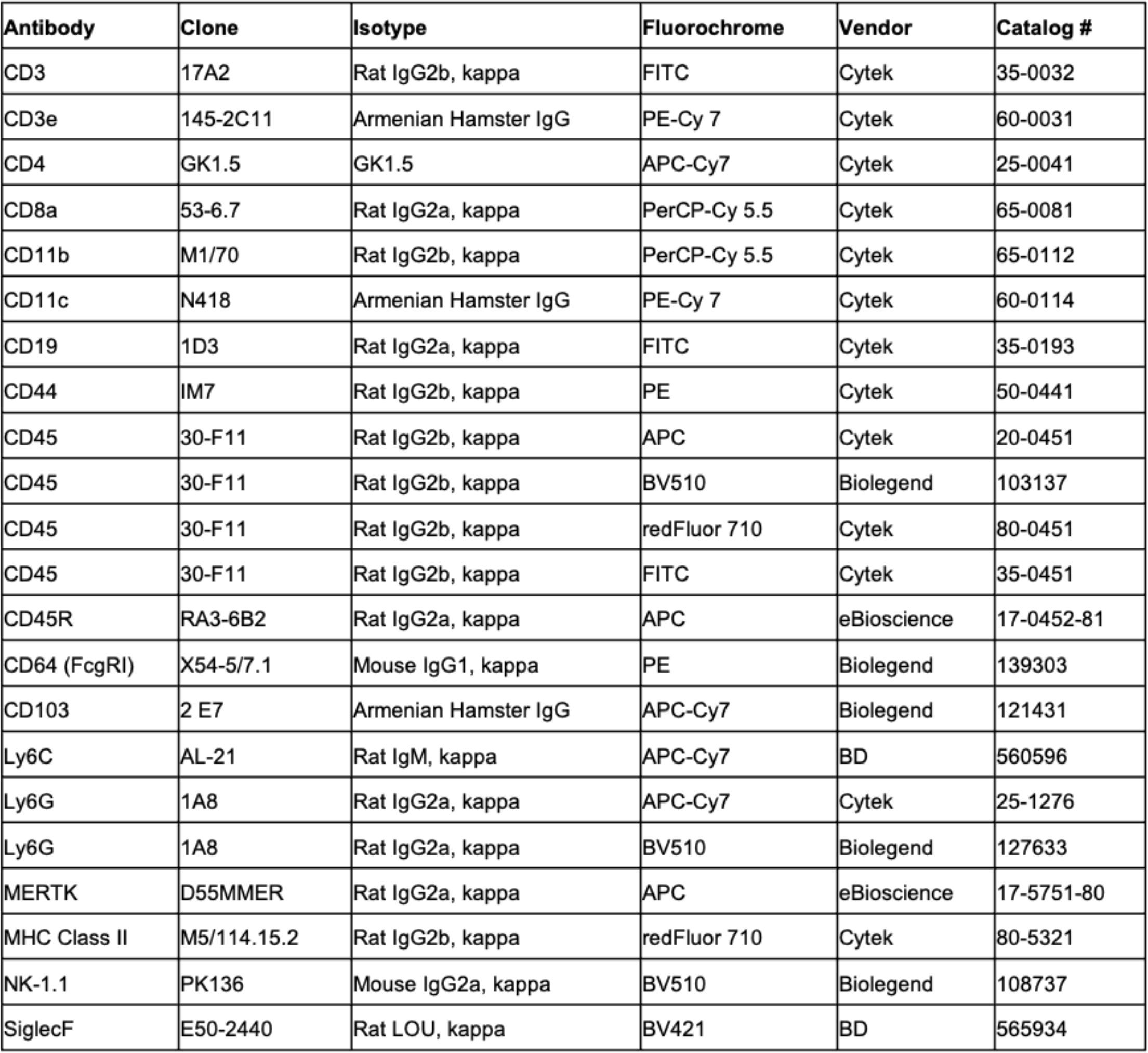

Supplement: S1 Table — (TIF) [file ppat.1013418.s011.tif]
